# Structure-dependent thermochromism of PAZO thin films: theory and experiment

**DOI:** 10.3762/bjnano.17.12

**Published:** 2026-01-20

**Authors:** Georgi Mateev, Dean Dimov, Nataliya Berberova-Buhova, Nikoleta Kircheva, Todor Dudev, Ludmila Nikolova, Elena Stoykova, Keehoon Hong, Dimana Nazarova, Silvia Angelova, Lian Nedelchev

**Affiliations:** 1 Institute of Optical Materials and Technologies “Acad. J. Malinowski”, Bulgarian Academy of Sciences, 1113 Sofia, Bulgaria; 2 Faculty of Chemistry and Pharmacy, Sofia University “St. Kliment Ohridski”, 1164 Sofia, Bulgaria; 3 Electronics and Telecommunications Research Institute, 218 Gajeong-ro, Yuseong-gu, Daejeon 34129, Republic of Korea

**Keywords:** aggregate, azopolymer, birefringence, PAZO, thermochromism

## Abstract

Poly[1-[4-(3-carboxy-4-hydroxyphenylazo)benzenesulfonamido]-1,2-ethanediyl, sodium salt] (PAZO) exhibits a range of unique physical properties that are critical for its diverse applications in photonics, optoelectronics, memory devices, and sensing technologies. In this study, we investigate the thermochromic behavior of PAZO thin films, focusing on the relationship between the structural organization of the polymer side chains and temperature-induced optical changes. By combining experimental spectroscopic techniques with theoretical modeling, we demonstrate that the thermochromic response of PAZO films is strongly influenced by molecular aggregation, film thickness, and thermal treatment conditions. The observed changes in optical properties suggest that this response is governed by temperature-induced modulation of molecular ordering and aggregation state, which in turn alters the electronic transitions responsible for light absorption. Theoretical calculations further support these findings, indicating that temperature-dependent intermolecular interactions and conformational changes play a significant role in shaping the optical behavior of the films. These results provide new insights into the structure–property relationships underlying thermochromism in azopolymer thin films and offer valuable guidelines for the design of thermally responsive photonic materials.

## Introduction

Functionalization of polymers with different photo- and bioactive groups to achieve novel physicochemical, optoelectronic, and even biophysical properties creates myriad opportunities for their application, especially as polymer thin films [1–2]. These films are widely used in modern life as they can be easily tailored to have specific properties like high conductivity, optical transparency, or chemical resistance, making them ideal for specialized applications [3]. Their cost-effectiveness, lightness, flexibility, and unique physical and chemical characteristics make them suitable for a wide range of industry applications including energy, optics, sensors, and microelectronics. The properties of polymers in thin films are known to markedly differ from those observed in the bulk, with significant differences in glass transition behavior, diffusion, and viscoelastic properties [4]. The assembly of polymer molecules from solution to solid state play a critical role in determining thin film morphology and properties. Polymer molecules can assemble through various intermolecular interactions, forming different aggregate structures that can drastically change the properties of solid-state materials. The wide variety of polymer chain types and architectures based on very diverse chemistries further extends the functionality of polymer thin films.

A specific group of photoreactive polymers comprises polymers with azo chromophores embedded in their structure [5–6]. These polymers exhibit photoresponsivity due to the *trans*–*cis* (*E*–*Z*) isomerization of the azo chromophores upon light irradiation. The isomerization leads to various effects, including molecular orientation, formation of surface relief structures, and photomechanical deformations. Consequently, azo polymers have been intensively studied as interesting photoresponsive materials [7–11].

**Figure 1:**
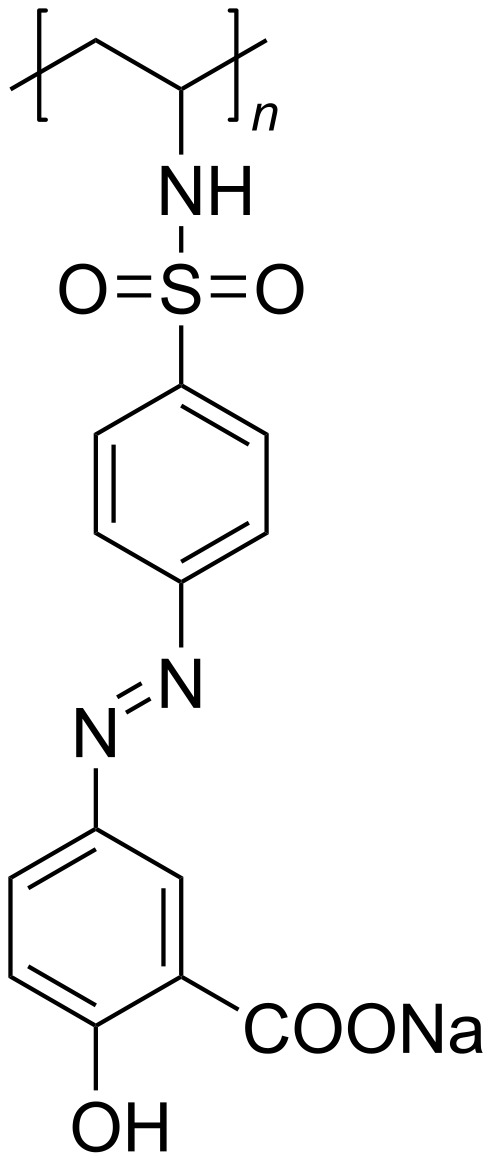
Chemical structure of the PAZO polymer.

One of the widely studied azo polymers is the commercially available poly[1-[4-(3-carboxy-4-hydroxyphenylazo)benzenesulfonamido]-1,2-ethanediyl, sodium salt], usually denoted as PAZO (Figure 1).

PAZO possesses a number of unique physical properties, most notably photoinduced birefringence – which are critical for its diverse applications in photonics, optoelectronics, memory devices, and sensors [12]. The birefringence phenomenon results from multiple cycles of *trans*–*cis*–*trans* (*E*–*Z*–*E*) photoisomerization stimulated by the polarized light impact [13–16]. The *E*–*Z*–*E* transitions lead to an alignment of the photoreactive azo chromophores perpendicularly (⟂) to the polarization direction of the electric field vector of the incident polarized light, resulting in a subsequent creation of birefringence.

Photoinduced birefringence of PAZO has been the subject of numerous studies under different experimental conditions. Investigations have been carried out on nondoped thin films [17–18], as well as on thin nanocomposite layers doped with nanoparticles of diverse chemical compositions, shapes, and sizes [19]. Additional efforts have focused on thermally treated layers of pure PAZO up to 200 °C [20], and on PAZO/PAH (with PAH denoting poly(allylamine hydrochloride)) layer-by-layer films examined over a range of temperatures [14,16]. Collectively, these studies highlight that the magnitude and stability of birefringence are strongly influenced by the supramolecular organization of the polymer chains. Despite these advances, a systematic understanding of how the molecular assembly governs birefringence in PAZO remains incomplete, leaving open questions about the fundamental mechanisms driving the observed photoresponsive behavior. This knowledge gap serves as the primary motivation for the present study. Here, we investigate the optical properties of PAZO thin layers subjected to thermal treatment at temperatures up to 300 °C. Particular attention is given to the changes in absorption features and spectral shifts that may arise from structural rearrangements within the material. To rationalize the experimental findings, density functional theory (DFT) calculations were employed to model possible supramolecular architectures, allowing us to explore the correlation between thermally induced optical responses and the underlying molecular-level organization.

## Results and Discussion

### Experimental results

#### Temperature dependence of absorption spectra

**Figure 2:**
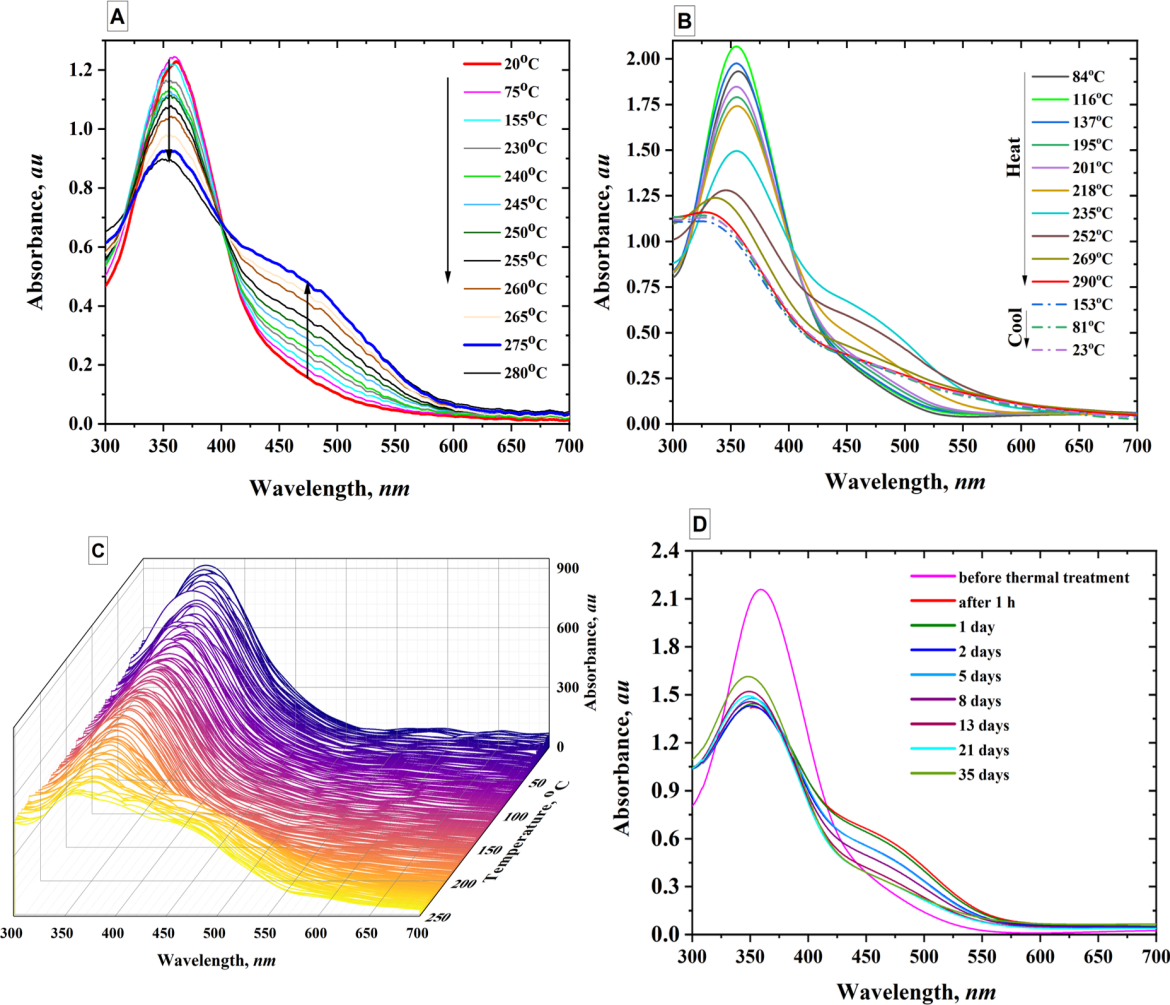
A) Absorbance at various temperatures (from r.t. to 280 °C, heating rate 5 °C per min); B) Absorption spectra, heating to 300 °C, heating rate 1 °C per min; C) Three-dimensional graph with temperature, wavelength and absorption as the three axes; D) Dependence of absorption spectra on time after stopping thermal treatment.

Absorption spectra for PAZO thin films at different temperatures were recorded upon heating as the temperature of the sample heater was gradually increased from room temperature (r.t.) to 300 °C (Figure 2A) at a rate of 5 °C/min and (Figure 2B) at a rate of 1 °C/min. The absorbance in the 450–550 nm region gradually increases with the rise of temperature up to 280 °C (Figure 2A), while the peak around 360 nm gradually decreases. Figure 2B shows two processes, one occurring at a lower temperature and the other within the 280–300 °C range.

For the first spectrum, shown in Figure 2B, the temperature is about 84 °C. So far, there is still no significant change in the spectrum compared to that at room temperature. After heating above 84 °C the spectrum starts to change with the band around 450–550 nm increasing until a critical temperature (≈230 °C) is reached, after which further heating no longer increases the absorption but rather starts decreasing it. Thus, the spectrum at 290 °C has lower absorption in this spectral region compared to the spectra at 269 °C and 252 °C. Simultaneously, the peak around 360 nm starts to shift to higher energies (hypsochromic shift), so we consider these new changes in the spectrum to be a new phase of PAZO transformation. Finally, the last three spectra in Figure 2B show how after reaching the maximum heating temperature of 300 °C and subsequent cooling to r.t., the spectrum remains relatively unchanged for a short time. It should be noted that the polymer retains its ability to undergo photoinduced isomerization in the temperature range of 280–300 °C, even though some chemical changes cannot be completely ruled out. According to differential thermal (DTA) and thermogravimetric (TGA) analyses, PAZO remains chemically stable up to about 280 °C, with indications of decomposition appearing only at higher temperatures (Figure S1, Supporting Information File 1). Figure 2C shows a three-dimensional plot with temperature, wavelength, and absorption as the three axes. From Figure 2C, the absorption dependences at selected wavelengths as a function of the heater temperature can be derived. Figure 2D tracks the changes in the spectrum as a function of time after the thermal procedure is completed. It can be seen that even after 35 days, the spectrum does not return to that taken before heating; on the contrary, it remains very similar to that taken 1 h after the heating stops. This is an indication that the structural changes in PAZO thin film induced by heating are significant and stable over weeks and even months.

These changes in the absorption spectra can be ascribed to the formation of aggregates between adjacent azochromophores – the so-called J-aggregates and H-aggregates, which will be discussed in more details in the Section “Density functional theory modeling”.

#### Analysis of spectrofluorimetric, infrared, and S and P polarization spectra

**Figure 3:**
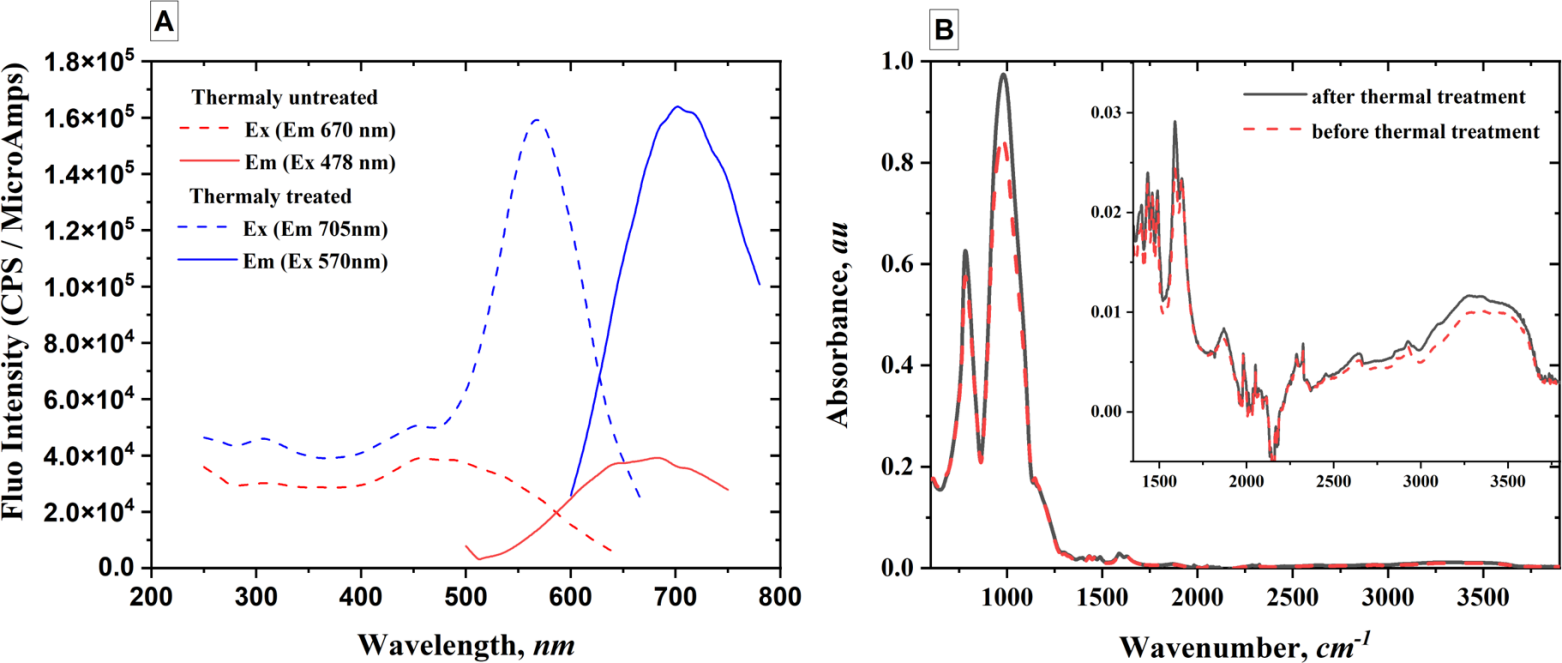
(A) Excitation spectrum (dashed line) and emission spectrum (solid line); (B) IR spectrum of PAZO before and after thermal treatment.

Two PAZO samples were used for the spectrofluorimetric analysis: the first sample was not thermally treated (red curves in Figure 3A) and the other was thermally treated in an oven to reach 200 °C (blue curves in Figure 3A). Spectrofluorimetry is a sensitive analytical technique that leverages fluorescence, where molecules absorb light and re-emit it at a longer wavelength. The process involves exciting a sample with a specific wavelength and measuring the intensity of the emitted fluorescence light at another wavelength. Treating PAZO at 230 °C induced an increase in fluorescence intensity (≈400%) and a redshift (20 nm). Red shift can be induced by various factors, such as changes in the polymer structure, aggregation, or interactions with other molecules (the interactions between polymer chains can be considered as major factors in the case of a polymer thin film).

Infrared (IR) spectroscopy is a powerful tool for understanding the effects of thermal treatment on polymers that can cause significant changes in their IR spectra, revealing alterations in molecular structure and chemical composition. Thermal treatment can alter the types and amounts of functional groups present in a polymer. This is a consequence of the chemical reactions occurring at elevated temperatures and leading to changes in the structure and properties of polymers. These reactions can involve the breaking of existing bonds within the polymer, the formation of new bonds, and the elimination or rearrangement of functional groups. In some cases, thermal treatment can induce crosslinking reactions, where polymer chains become interconnected, leading to increased stiffness and improved mechanical properties. Figure 3B shows the IR spectra of PAZO samples before and after thermal treatment. There are no drastic changes in the band positions, but some changes in the band intensities are observed. Variation of the band intensities with temperature increase for absorption bands appearing at ≈975 cm^−1^, 1590 cm^−1^, and in the range of 2500–3500 cm^−1^, are observed. These observations do not provide enough data to determine the exact changes produced in the polymer chain during thermal treatment, but some oxidation processes cannot be completely ruled out.

**Figure 4:**
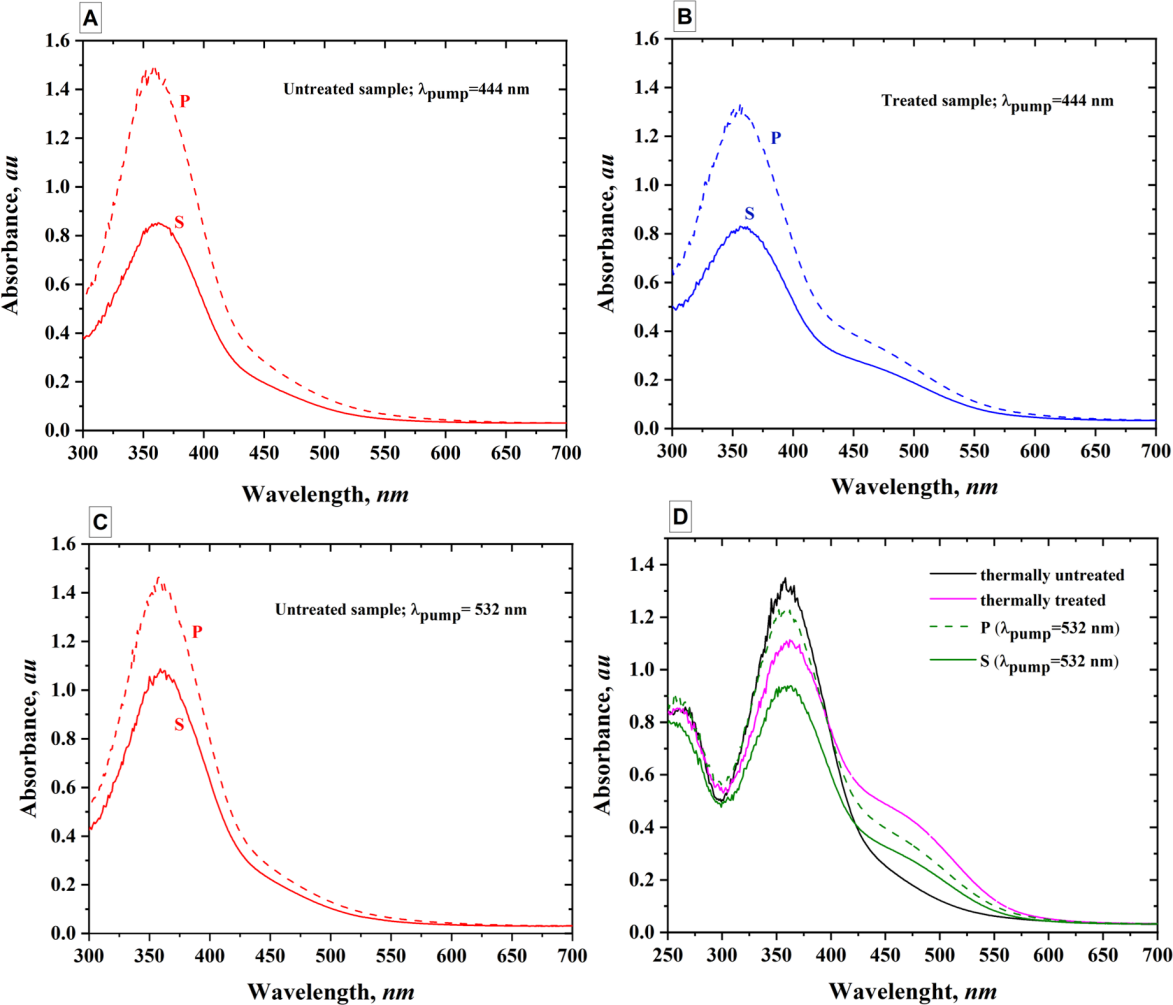
S and P polarization spectra for PAZO illuminated with pump laser at 444 nm (A and B) and 532 nm (C and D). Thermally untreated (A and C) and thermally treated for 1 h at 200 °C (B). Comparison between the PAZO sample, thermally treated sample, and S and P polarization spectra for illuminated samples (D).

As mentioned in the Experimental and Computational Details section, a CARY spectrophotometer was used to measure the absorption spectra under S- and P-polarized light (where P-polarized light has its electric field vector parallel to the plane of incidence, and S-polarized light is perpendicular to this plane). Four samples were prepared for the study. Two of them underwent thermal treatment at 200 °C for 1 h, resulting in the emergence of a new absorption band with a peak in the 460–475 nm range. Birefringence was then induced in pairs of samples using 444 and 532 nm laser illumination. Following sample treatment and laser illumination, S- and P-polarized absorption spectra were recorded for each case. The results, presented in Figure 4A–D, reveal the polarization sensitivity of the samples under four distinct experimental conditions. As it can be seen, substantial spectral change in P and S spectra are observed for the sample under laser illumination at 532 nm. To emphasize these changes, we plotted in the Figure 4D the spectra of the untreated PAZO sample and the thermally treated one.

Quantitative interpretation of the obtained P and S absorption spectra can be done by calculating an order parameter, *S*_order_, from the following expression: *S*_order_ = (*A*_∥_ − *A*_⟂_)/(*A*_∥_ + 2*A*_⟂_), with *A* standing for the measured absorption and the indices show whether the polarization of the light is parallel or perpendicular to the polarization of the pump light [21]. This order parameter characterizes the level of organization of the molecules after irradiation with polarized light. Before inducing birefringence in the samples, *S*_order_ = 0 because the values of *A*_∥_ and *A*_⟂_ are equal. The molecules are randomly oriented. After inducing birefringence, a higher value of the order parameter is an evidence of higher anisotropy. Table 1 presents the values of *S*_order_ calculated at 360 and 460 nm. These wavelengths are chosen as corresponding to the two peaks of absorption for the thermally treated samples. Comparison of the *S*_order_ values for the treated and untreated samples indicates that the untreated sample exhibits more favorable absorption characteristics under illumination at 444 nm, while the thermally treated sample shows better absorption efficiency under illumination at 532 nm.

**Table 1:** Calculated values of the *S*_order_ parameter for the two peaks of absorption at 360 and 460 nm.

	Thermally untreated sample			Thermally treated sample		
		
Wavelength, nm	*A*_∥_	*A*_⟂_	S_order_	*A*_∥_	*A*_⟂_	*S*_order_

Pump laser wavelength 444 nm						

360 nm	1.49	0.85	0.55	1.29	0.83	0.44
460 nm	0.25	0.17	0.36	0.36	0.27	0.30

Pump laser wavelength 532 nm						

360 nm	1.45	1.09	0.29	1.22	0.94	0.39
460 nm	0.24	0.20	0.20	0.37	0.31	0.34

#### Birefringence (∆*n*) before and after thermal treatment

**Figure 5:**
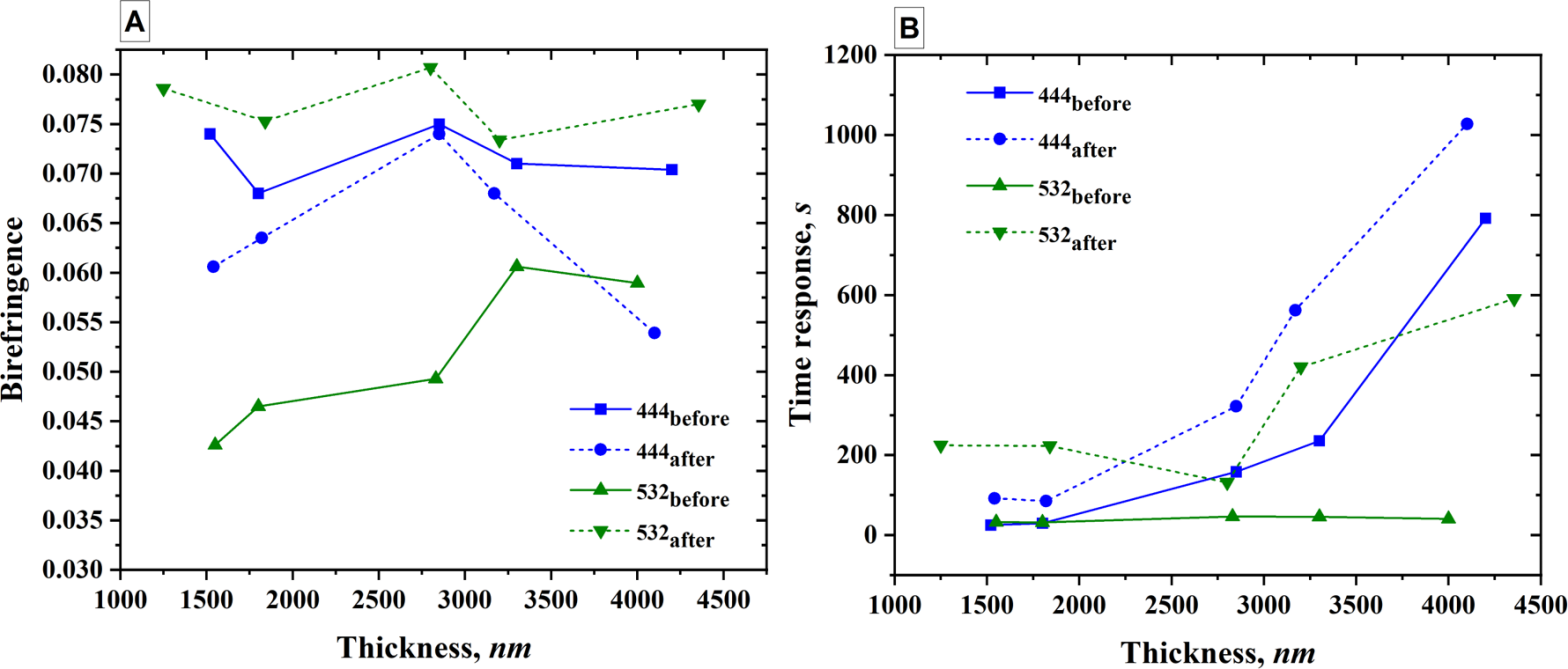
A) Dependence of ∆*n* on the thickness of the sample before and after thermal treatment using an oven and two different pump lasers at 444 and 532 nm. B) Dependence of the response time on the thickness of the sample before and after thermal treatment.

For the measurement of ∆*n* at 444 and 532 nm, we prepared different samples with the thicknesses in the range of 1200–4000 nm on a glass substrate. Thicker films were prepared because many potential applications – such as the inscription of polarization-selective holographic optical elements – require greater film thickness for optimal performance. The samples were heated in an oven and the measurement of ∆*n* started approximately 1 h after the samples had cooled down to r.t. The corresponding data are presented in Figure 5.

From the results it is evident that thermal treatment does not enhance the birefringence (∆*n*) when the pump laser wavelength is 444 nm. In contrast, a significant improvement is observed when a 532 nm laser is used. Additionally, the thermal treatment leads to an increase in the response time in both cases. These findings can be analyzed in the context of previously reported results. In the study by Nedelchev et al. [18], a spectral dependence of ∆*n* on the pump laser wavelength was reported, which provides further insight into the observed behavior.

Analysis of the spectra obtained from the treated samples reveals the formation of aggregates, in agreement with previously reported observations [21]. Furthermore, the observed increase in birefringence within the newly formed absorption band suggests that these aggregates undergo reorientation upon exposure to polarized light. Spectrofluorimetric measurements indicate that a significantly higher energy input is required to induce molecular reorientation, as a substantial portion of the energy from the pump laser is dissipated through fluorescence. Moreover, the reorientation of the aggregates themselves appears to demand an even greater energy threshold. The enhancement of absorption around 444 nm is of limited practical utility, as the absorption becomes excessively strong, restricting effective energy delivery to the surface layers of the sample. Conversely, the increased absorption at 532 nm is advantageous, offering sufficient absorption while allowing deeper light penetration without significant energy loss. This facilitates the efficient use of high-power lasers operating at 532 nm for the fabrication of diffraction gratings with enhanced efficiency or expanded surface coverage.

#### Density functional theory modeling

**Figure 6:**
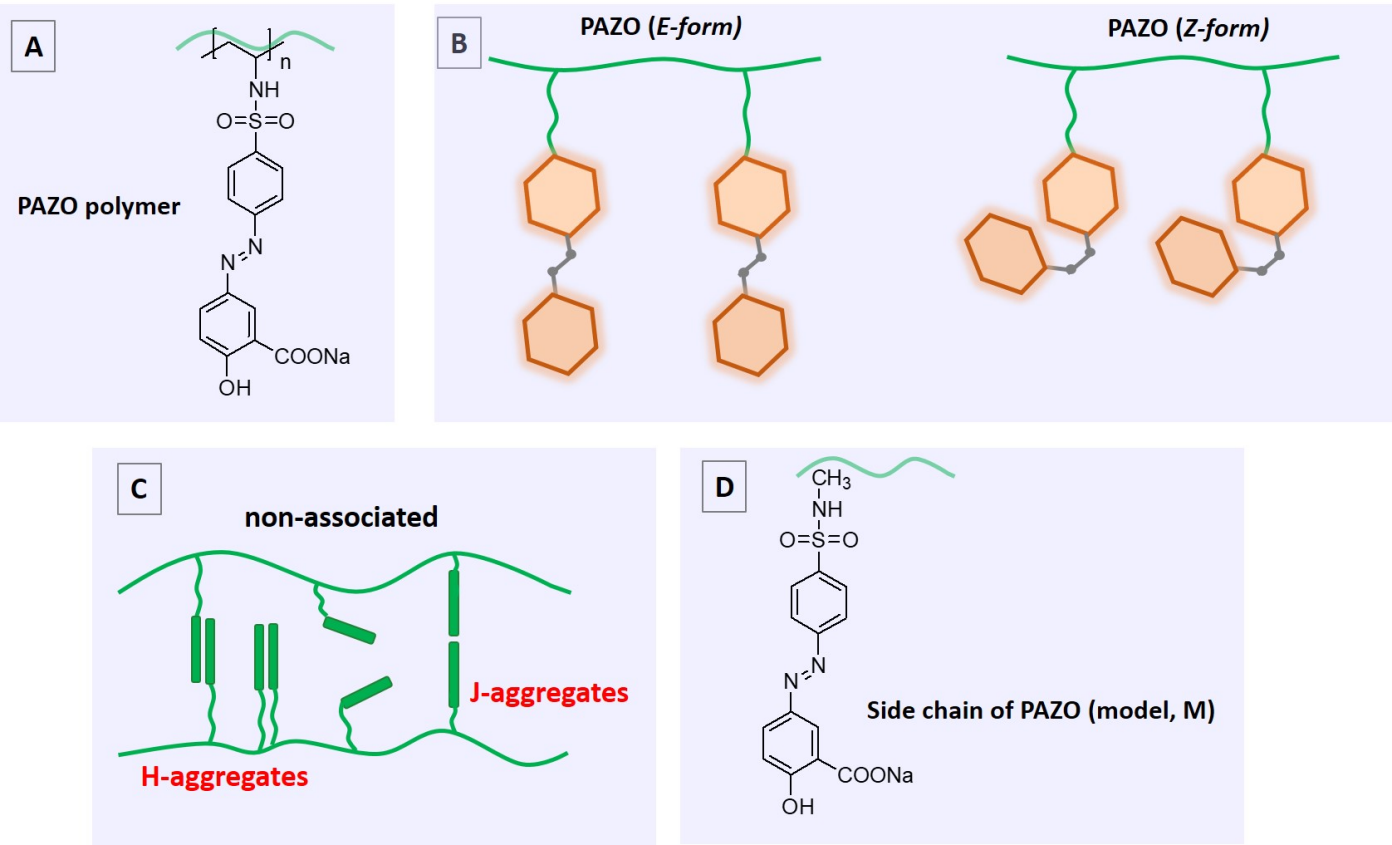
A) Chemical structure of the PAZO polymer; B) Schematic presentation of *E-* and *Z-*forms of the PAZO polymer side chains. *E*–*Z notation is* employed in our study for naming the azo group containing simplified models of the side chains of PAZO (this nomenclature is preferred since it describes absolute configuration, whereas *cis*–*trans* merely describes relative configuration); C) Schematic representation of nonassociated and associated side chains of two PAZO polymer chains; D) A simplified model of a PAZO side chain.

PAZO is a polymer with azobenzene units incorporated as pendant groups attached to the main polymer backbone (Figure 6A). The core of PAZO polymer side chains is the azobenzene fragment (Ar–N=N–Ar), which can exist in two isomeric forms: a stable *trans* (*E*) form and a metastable *cis* (*Z*) form (Figure 6B). The self-assembly of PAZO polymers into various nanostructures is mainly driven by the noncovalent interactions, such as π–π stacking between azobenzene groups. Azobenzene groups are photosensitive and can undergo reversible *E*–*Z* isomerization upon exposure to light with different wavelengths. This *E*–*Z* isomerization of the azobenzene units leads to significant changes in the conformation, supramolecular structure, and bulk properties of the polymer such as optical anisotropy, solubility, and mechanical behavior.

Тhe properties of a photoreactive polymer can be largely influenced by the aggregation structures and the molecular stacking models. The organization of the polymer chains (main and side) can be extremely complex both in solution and in the aggregated state. Recent studies on a polymer similar to PAZO have shown a complex hierarchical chiral arrangement [22]. The study of Peiru Min et al. concluded that limited *E*–*Z* isomerization of azobenzene pendants in side-chain homopolymers leads to a higher concentration of the *E*-isomer in the photostationary state. This increase in *E*-isomers promotes stronger noncovalent π–π interactions between the azobenzene groups, ultimately driving the self-assembly of these polymers into spherical micelles [23].

**Figure 7:**
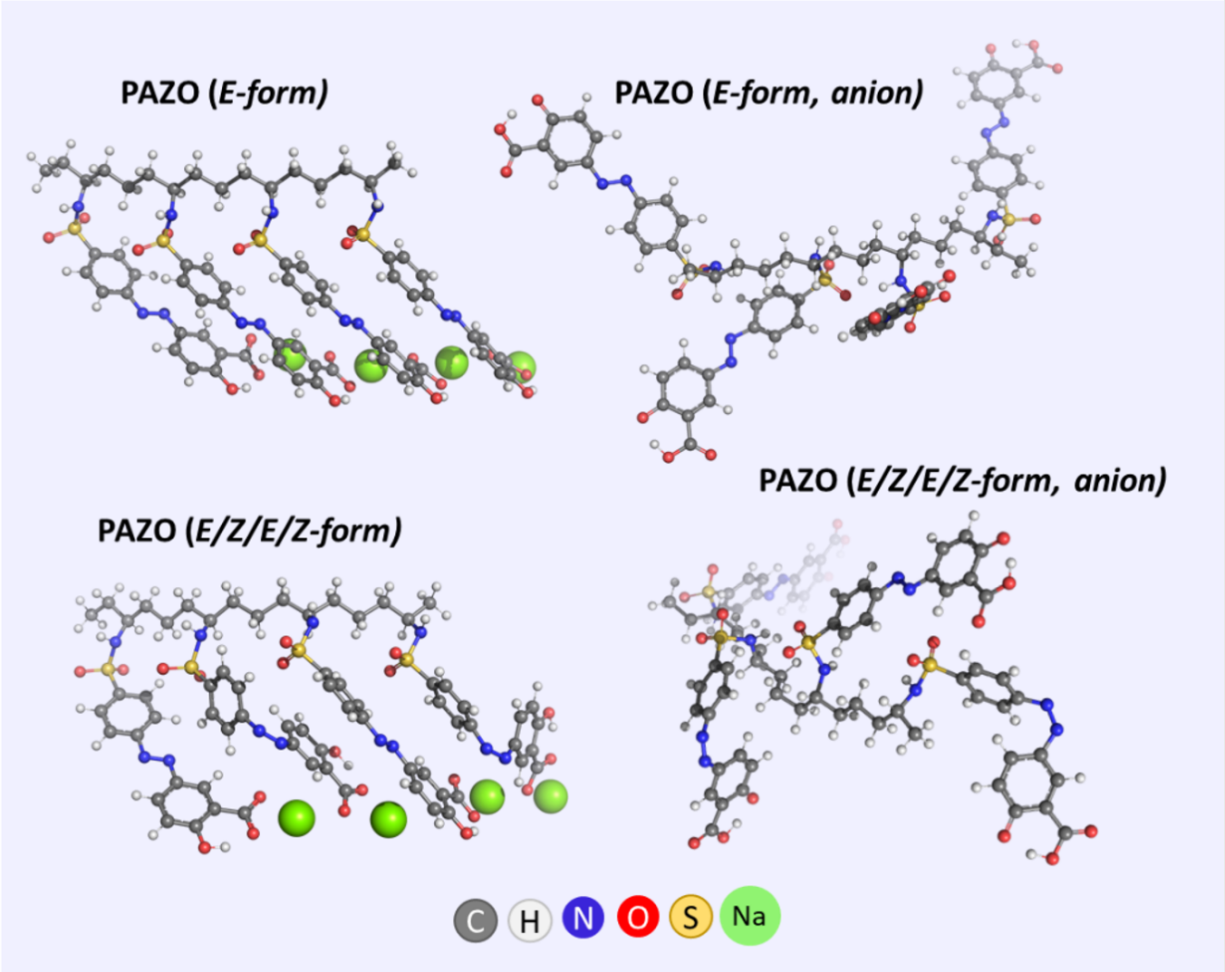
Optimized models of fragments of the polymer main chain (different forms, with/without counterion) with four side chains.

An important characteristic of photoreactive polymers is that not only chromophores undergo reorientational motion, but also entire polymer segments rearrange upon external impact [16,24]. It is known that photoinduced birefringence in thin films containing azobenzene derivatives is generated by the orientation of chromophores in response to repeating cycles of *E*–*Z*–*E* photoisomerization [15]. After a given photoisomerization cycle, chromophores can be oriented in any direction, and those whose dipole moment is perpendicular to the polarization direction of the writing laser will no longer undergo photoisomerization. After a series of cycles, there will be a greater number of chromophores perpendicular to the direction of polarization than in any other direction, resulting in birefringence. The ability of chromophores to isomerize and reorient is strongly influenced by the free volume (unoccupied space) available within a material. The free volume (critical volume) (i.e., the space available for molecular motion within a material) is influenced by both temperature and molecular interactions. As temperature increases, thermal motion increases, leading to a larger free volume. Conversely, stronger molecular interactions can restrict the free volume, effectively creating a smaller space for molecules to move around. In an attempt to study the aggregation of the PAZO polymer with a relatively accurate but computationally expensive method (DFT calculations), models of a fragment of the polymer main chain with four side chains were considered (Figure 7).

PAZO is a polymer with ionized end groups and the orientation of PAZO chromophores is highly dependent on the presence of counterions. A comb-like structure of PAZO is observed in the presence of positively charged sodium ions close to –COO^−^ groups, while in the absence of counterions, the side chains do not form an ordered structure (Figure 7). It can be concluded that counterions need to be taken into account to adequately simulate the cooperative aggregation of PAZO side chains. The photoisomerization of the azobenzene groups in the polymer plays a crucial role – upon exposure to ultraviolet light, the azobenzene groups undergo *E*–*Z*–*E* photoisomerization, leading to configuration differences in the polymer side chains. A comb-like ordered structure is observed in both modeled all-*E* and *E/Z/E/Z* fragments of the polymer main chain with four side chains (Figure 7).

**Figure 8:**
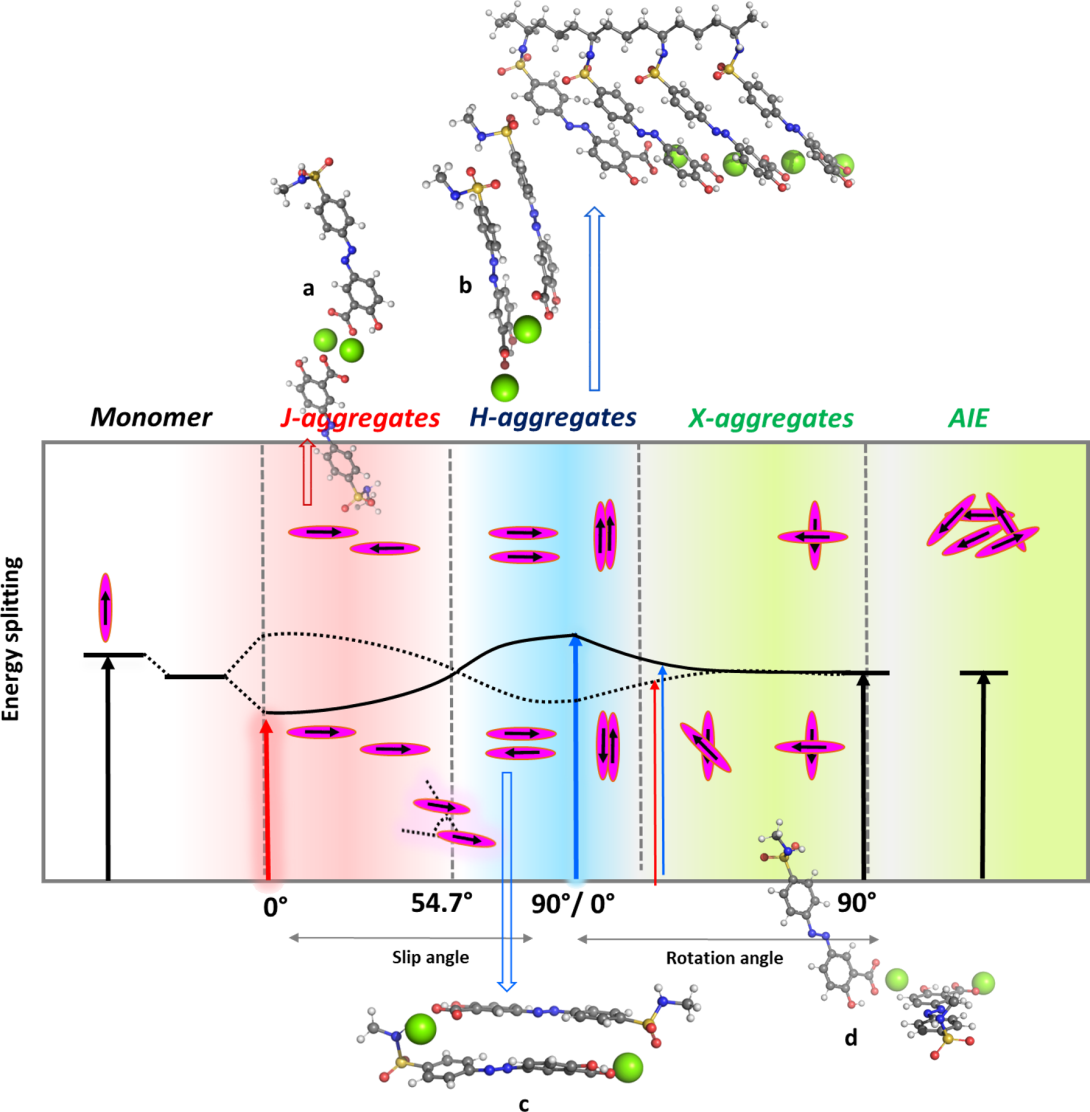
Schematic representation of different molecular packing patterns, orientation of the chromophores and resulting change in the optical properties of the isolated monomers. The possible dimers formed by the *E*-forms of the simplified PAZO side-chain model (shown in Figure 6D) are also presented in Figure 8.

PAZO is a side-chain azo polymer, and the distances between the pendant azo groups and the main chain, as well as between the side chains themselves, also affect the properties of the polymer. From the optimized geometries of the all-*E* and *E/Z/E/Z* fragments of the polymer main chain with four side chains, it can be seen that the *all-E* form has the intrinsic ability to form an ordered structure due to the strong noncovalent π–π interaction between the chromophores (Figure 7). Side chains from different main chains of the polymer can interact in a similar way to form supramolecular aggregates (Figure 8) [22]. The π-stacked aggregates are usually classified as H- and J-aggregates, and this classification (originally worked out by Kasha) is based on the optical properties of the aggregates arising from the different molecular packing patterns. H-aggregates are usually characterized by side-by-side (cofacial) “sandwich” orientations of the monomeric chromophores, and the main absorption peak is generally blueshifted (“H” for hypsochromic). Conversely, neighboring chromophores, arranged in a head-to-tail fashion, give a negative value for the Coulomb coupling and lead to the occurrence of aggregation-induced redshift (they are called “J”-aggregates after E.E. Jelley, who discovered the phenomenon in 1936).

Packing geometries in the aggregate state actually include a variety of molecular aggregate structures ranging from “HJ” dimers to more complex formations including bent, herringbone, and chiral aggregates [25–26]. Figure 7 illustrates the different molecular packing patterns, the orientation of the chromophores, and the expected change in the optical properties of the isolated monomers. The possible dimers formed by the *E*-forms of the simplified PAZO side-chain model (shown in Figure 6D) are also presented in Figure 8.

**Figure 9:**
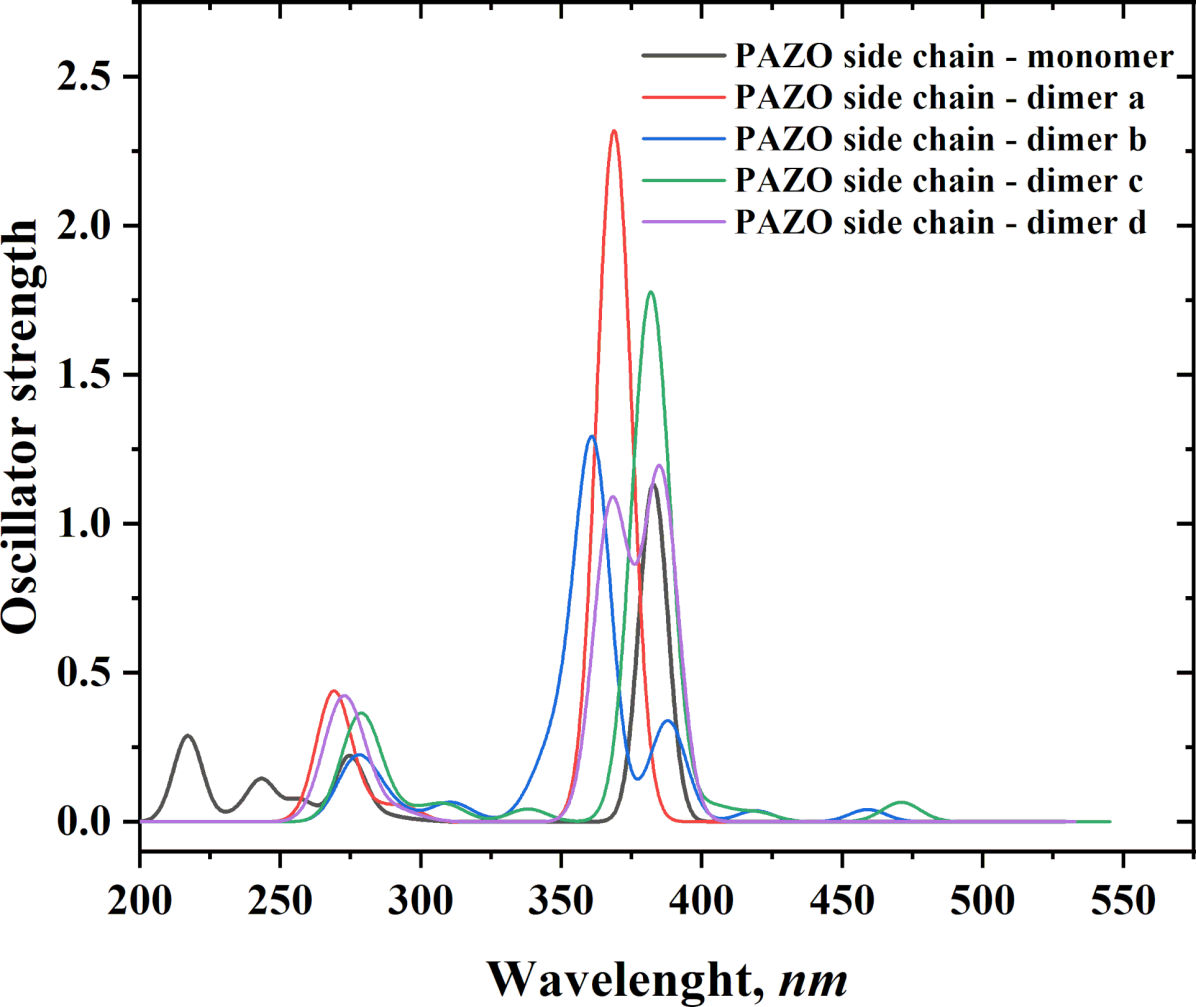
PBE1PBE/6-311+G(2d,p) simulated spectra of PAZO side chain monomer and dimer a–d; Gaussian broadening is applied: band width on 1/2 height: 0.15 eV.

The aggregation upon heating hypothesis can be confirmed by simulating the absorption spectra of monomers and dimers of the chromophore containing azobenzene and comparing them with experimental ones. The time-dependent DFT (TDDFT) scheme was used to calculate at PBE1PBE/6-311+G(2d,p) level of theory the absorption spectra of the simplified models of nonaggregated/aggregated PAZO side chains starting from the ωB97XD/6-31G(d,p) optimized structures in solvent chloroform (used to mimic the polarity of the polymer layer). Simulated (using the vertical excitation energies and oscillator strengths) UV spectra are given in Figure 9. One strong monomer band at 383 nm is predicted. The spectra of the model dimers clearly differ from the monomer spectrum, with various effects observed, including bathochromic, hypsochromic, hyperchromic, and hypochromic shifts of the monomer band. For example, aggregate formation leads to the appearance of low intensity bands (dimers c and d) in the 400–480 nm range. The hypsochromically shifted bands of the a–d dimers are quite intense, but due to the dynamic nature of the aggregates formed between the PAZO side chains, it can be assumed that an equilibrium between different short-lived aggregates is present. Тhe band positions and the splitting that is predicted from the simulated absorption spectra upon azobenzene fragments dimer/aggregate formation qualitatively explains the experimental absorption spectra of PAZO.

It should be noted that aggregates formed between side chains of one or two polymer chains cannot be classified as clear H-, J-, X-aggregates. The performed calculations at r.t. for dimer formation reactions reveal that, from the thermodynamic point of view, the formation of a–d dimers is possible, the reactions are characterized by negative values of Gibbs energies ranging from −24.3 to −9.3 kcal·mol^−1^ in the gas phase, and from −10.8 to −3.7 kcal·mol^−1^ in chloroform (Table 2).

**Table 2:** ∆*G*^ε^ values (in kcal·mol^−1^) for the reactions of formation of dimers a–d. The upper index indicates results in the gas phase (ε = 1) and in chloroform (ε = 5) surroundings, yielded at ωb97XD/6-31+G(d,p)//ωb97XD/6-31G(d,p) level of theory.

Reaction	25 °C		200 °C	
	
	Δ*G*^1^	Δ*G*^5^	Δ*G*^1^	Δ*G*^5^

2*M → dimer "a"	−21.7	−8.3	−15.0	−1.5
2*M → dimer "b"	−15.0	−8.3	−5.4	1.4
2*M → dimer "c"	−24.3	−10.8	−13.0	0.6
2*M → dimer "d"	−9.3	−3.7	−3.6	2.1

It should be noted that dimer formation is favored both between adjacent azobenzene fragments within a single main polymer chain and between fragments belonging to distinct polymer chains. At elevated temperatures, aggregation was observed almost exclusively in nonpolar environments (ε = 1). This finding highlights the influence of solvent polarity, as the diminished dielectric screening characteristic of nonpolar media enhances monomer–monomer association, whereas polar environments preferentially stabilize the monomeric state.

## Conclusion

Understanding the aggregation states and thermal molecular motion of polymer chains in thin films is essential for tailoring the properties and enhancing the performance of polymer-based devices. By precisely controlling these factors, researchers can fine-tune the structure and dynamics of the film to achieve specific characteristics suited for a wide range of applications. This knowledge is particularly important in fields such as organic electronics and drug delivery, where thin films play a pivotal role. Our study focuses on these aspects to deepen the understanding of how to optimize PAZO thin films for photonic and optoelectronic applications. We investigated the thermochromic behavior of PAZO films and highlighted the significant impact of structural organization and thermal treatment on their optical properties. Thermal annealing notably enhanced the photoinduced birefringence under 532 nm laser excitation, while no such improvement was detected at 444 nm. Moreover, thermal treatment resulted in longer response times, likely due to alterations in molecular ordering and aggregation. By integrating experimental observations with theoretical analysis, this work provides valuable insights into structure–property relationships that govern thermochromism and polarization sensitivity in azopolymer thin films. These findings advance the rational design of thermally and optically responsive materials with promising potential in photonics and optoelectronics.

## Experimental and Computational Details

All thin films used in this study were based on PAZO polymer.

### Preparation of thin-film samples

All thin films used in this work were fabricated using spin-coating technique. Methanol was used as solvent for all samples, and a magnetic stirrer ensured complete dissolution. The solution was then poured onto the substrate (quartz or glass) and spun at 1000 rpm to achieve thin film thicknesses ranging from 200 to 4000 nm. For spectral measurements, quartz substrates were used due to their lack of absorbance in the PAZO absorption region; thin film thickness was about 200 nm. A glass substrate was used for the measurement of birefringence (∆*n*) as being transparent to the wavelength of the lasers used; thin film thickness varied from 500 to 4000 nm. The film thickness was measured using a Filmetrics F20 high-precision thin-film analyzer. The samples were examined before and after the thermal treatment performed by using thermal stage THMS600 (Linkam Scientific). A Dentamatic furnace (model 600) was used to heat the glass-deposited layers for 1 h at 200 °C. To avoid changes in the absorbance over time that influence the measurements, the optical experiments were performed immediately after the samples were cooled down to r.t. after the thermal treatment. This ensured that the recorded spectra reflect the state of the material right after the treatment.

### Spectral measurements

A Varian Cary 5E UV–vis–NIR spectrometer was employed to analyze absorption spectra of samples (thickness 200–300 nm) in the 250–800 nm spectral range before and after thermal treatment. S and P polarization spectra were also measured after the birefringence measurements.

The ATR-IR (attenuated total reflectance IR) spectra were collected using a Thermo Nicolet iS50 FTIR spectrometer with a diamond crystal ATR accessory. The measurements were done at r.t. across a spectral range of 4440 to 600 cm^−1^ with resolution of 2 cm^−1^ and 64 scans used.

The fluorescence spectra were recorded using a FluoroLog 3–22 (HORIBA) spectrofluorometer in the spectral range of 250–800 nm with resolution of 0.5 nm and double-grating monochromators by excitation wavelength set near to the absorption peak of the films.

### Real-time absorbance measurement during the heating of the samples

**Figure 10:**
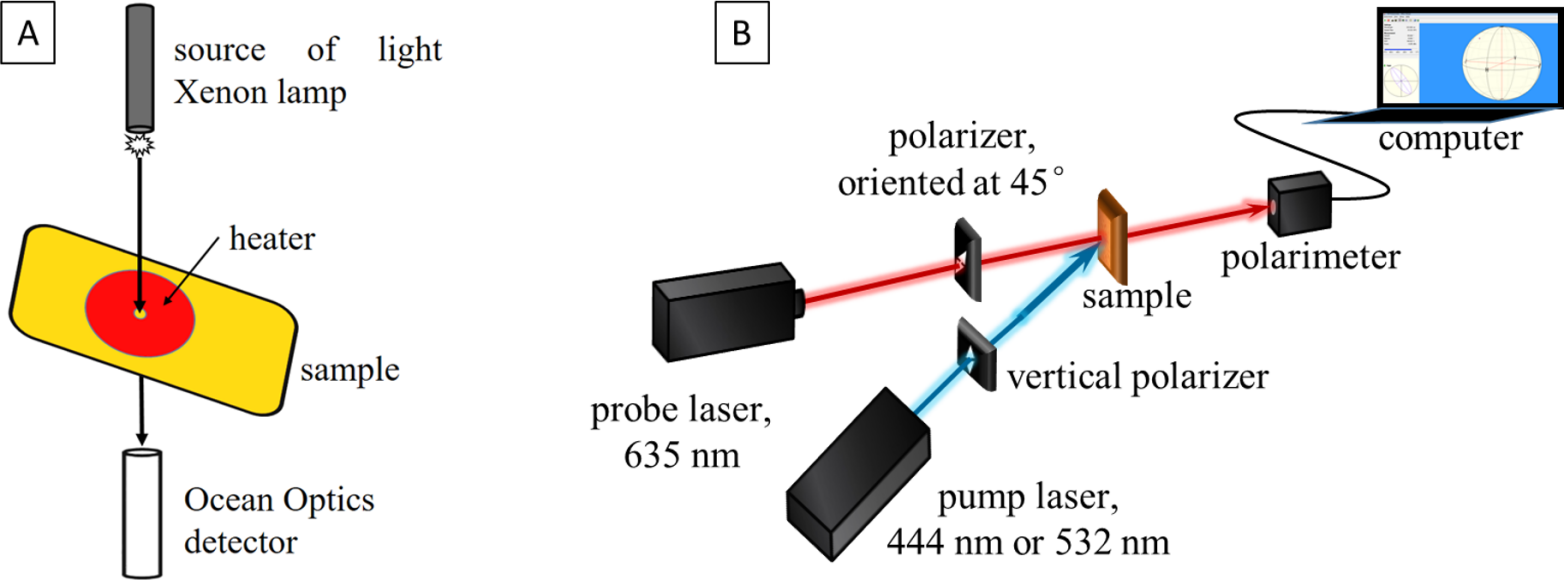
(adapted from [20] (© 2024 G. Mateev et al., published by EDP Sciences, distributed under the terms of the Creative Commons Attribution 4.0 International License, https://creativecommons.org/licenses/by/4.0)): Experimental setup for: (A) thermal heating and real-time absorbance measurements of the samples; (B) optical setup for birefringence measurement.

The control of temperature and subsequent thermal heating of the samples was achieved by using a THMS600 (Linkam Scientific) heating and freezing stage, which is capable of maintaining a given speed of heating with a 0.1° precision. Samples were heated from r.t. (20–25 °C) to 300 °C at two rates: of 5 °C/min and 1 °C/min. The system used allows for optical measurements of a small area of the sample, so the stage was mounted between the xenon lamp as a source of light (in the range 250–1000 nm) and the spectrophotometer (Ocean Optics) detector system (Figure 10А).

### Birefringence measurement

A classic setup with a polarimeter, as shown in Figure 10B, was used to measure birefringence. This setup included a light source, a polarizer, a birefringent sample, and a polarimeter to measure the polarization state after passing through the sample. Two lasers were used: the "pump" laser, which excites the sample, and the "probe" laser, which interacts with the sample to observe its changes after excitation. For pump lasers, we used a DPSS laser at 444 nm (Cobolt Calypso) and a powerful Verdi (Coherent) laser at 532 nm. A second DPSS laser at 635 nm was used as a probe laser. The sample, before and after thermal heating, was put in the crossing point of the pump and probe laser beams. The pump light had vertical polarization and intensity around 125 mW/cm^2^ for both pump lasers, while the probe light had a 45° linear polarization. The pump beam is partially absorbed by the polymer film and provokes reorientation of PAZO molecules perpendicular to the vertical polarization of the light, so the isotropic films become anisotropic. Using the polarimeter, we tracked the changes in polarization of the probe light. The latter was not expected to be absorbed by the PAZO even after the heating procedure since the changes in the absorption spectrum occured below 635 nm. All measurements of birefringence were performed one day after the heating procedure, so that conditions were the same for all samples.

### Computational details of DFT calculations

Molecular modeling tools were used in the current study to shed some light on the molecular structure and interactions (including photoinduced cooperative molecular reorientation) of PAZO that determine its distinct properties. PAZO is a polymer and its molecular weight can vary depending on the polymerization process and the specific PAZO sample, with some having an average weight of 50000 g/mol. Efficient and accurate DFT techniques [27] are able to predict the structure and properties of single- and multi-component systems. High-level DFT calculations with hybrid or meta-GGA functionals are desirable for geometry optimization, but considering a polymer with a realistic length would make the calculation too long and, therefore, expensive. On the other hand, the properties of the material (polymer), including crystallinity, density and solubility, are significantly affected by the side chains – the branches that extend from the main polymer backbone. These chains can be short (oligomeric) or long (polymeric), and their length and chemical nature determine the behavior of the polymer. Simplified models of long polymer side chains often represent well the structure and flexibility of the polymer. These models can be used to understand how the properties of the side chains affect the overall behavior of the polymer, including its ability to interact with other molecules. Applying this practical solution to the "realistic polymer system size" vs "computational cost" dilemma, we modeled a PAZO side chain.

The molecular geometries of the studied isomers and conformers of the modeled single PAZO side chain and fragments with up to four side chains were optimized at the ωB97XD/6-31G(d,p) level of theory. Since the noncovalent interactions calculated with DFT typically rely on empirical dispersion terms, a range-separated hybrid functional with built-in empirical dispersion correction, ωB97XD, was chosen for our study. It has been supposed that the repulsion of long-range exchange interactions plays a major role in weak bonds such as van der Waals bonds, and, therefore, long-range correction is the best strategy for enhancing the accuracy of the Kohn–Sham method in the calculations of weakly bonded systems [28]. The computations were performed with the 6-31G(d,p) basis set for all atoms [29–30]. The geometry optimization of each structure was followed by frequency calculations performed at the same level of theory. No imaginary frequency was found for the lowest energy configurations of any of the optimized structures. The vibrational frequencies were used to compute the thermal energies, *E*_th_, including zero-point energy, and entropies, *S*. The differences Δ*E*_el_, Δ*E*_th_, *P*Δ*V* (work term), and Δ*S* between the products and reactants were used to evaluate the gas-phase free energy of the supramolecular complex formation/aggregation, Δ*G*^1^, at *T* = 298.15 K according to:


[1] $$\Delta G^{1}=\Delta E_{\text{el}}+\Delta E_{\text{th}}+P\Delta V-T\Delta S.$$

Solvation effects were accounted for by employing the solvation model based on density (SMD) [31] as implemented in Gaussian 09 suite of programs [32]. Single-point calculations were performed at the ωB97XD/6-31+G(d,p) level in the gas phase (ε = 1) and in chloroform (ε = 5) for the corresponding structures optimized at the ωB97XD/6-31G(d,p) level (ωB97XD/6-31+G(d,p)//ωB97XD/6-31G(d,p)). Solvation free energies of the products and reactants were used to calculate the free energy of the supramolecular complex formation in chloroform following the standard thermodynamic cycle approach:


[2] $$\Delta G^{5}=\Delta G^{1}+\Delta G_{\text{solv}}^{5}\left(\text{Products}\right)-\Delta G_{\text{solv}}^{5}\left(\text{Reactants}\right).$$

A positive ∆*G*^ε^ (ε = 1 or 5) implies a thermodynamically unfavorable supramolecular complex formation/aggregation, whereas a negative value implies a favorable one. DFT calculations at 200 °C (473.15 K) were used to study molecular properties at this specific temperature. UV–vis spectra of a single PAZO side chain model and fragments/aggregates were calculated by TD-DFT at PBE1PBE/6-311+G(2d,p) level of theory.

The PyMOL molecular graphics system was used to generate the molecular graphics images [33].

## Supporting Information

File 1 Additional experimental data.

## Data Availability

All data that supports the findings of this study is available in the published article and/or the supporting information of this article.

## References

[1] Blasco E, Sims M B, Goldmann A S, Sumerlin B S, Barner-Kowollik C (2017). Macromolecules.

[2] Trefon-Radziejewska D, Juszczyk J, Opilski Z, Pawlak M, Hamaoui G, Powroźnik P, Smokal V, Krupka O, Derkowska-Zielinska B (2023). J Phys Chem C.

[3] Tsui O K C, Russell T P (2008). Polymer Thin Films.

[4] Jones R A L (1999). Curr Opin Colloid Interface Sci.

[5] Baroncini M, Groppi J, Corra S, Silvi S, Credi A (2019). Adv Opt Mater.

[6] Wang X, Wang X (2017). Introduction. Azo Polymers: Synthesis, Functions and Applications.

[7] Yu H (2014). J Mater Chem C.

[8] Wang D, Wang X (2013). Prog Polym Sci.

[9] Sun S, Liang S, Xu W-C, Xu G, Wu S (2019). Polym Chem.

[10] Todorov T, Nikolova L, Tomova N (1984). Appl Opt.

[11] Nikolova L, Ramanujam P S (2009). Polarization Holography.

[12] Liu Y, Shi K, Zhitomirsky I (2015). J Mater Chem A.

[13] Yang J, Zhang J, Liu J, Wang P, Ma H, Ming H, Li Z, Zhang Q (2004). Opt Mater (Amsterdam, Neth).

[14] Timóteo A R M, Ribeiro J H F, Ribeiro P A, Raposo M (2016). Opt Mater (Amsterdam, Neth).

[15] Delaire J A, Nakatani K (2000). Chem Rev.

[16] Ferreira Q, Ribeiro P A, Oliveira O N, Raposo M (2012). ACS Appl Mater Interfaces.

[17] Nedelchev L L, Nazarova D I, Petrova P (2013). Bulg Chem Commun.

[18] Nedelchev L, Ivanov D, Blagoeva B, Nazarova D (2019). J Photochem Photobiol, A.

[19] Nazarova D, Nedelchev L, Berberova-Buhova N, Mateev G (2023). Nanomaterials.

[20] Mateev G, Dimov D, Nazarova D, Stoykova E, Hong K, Nedelchev L (2024). EPJ Web Conf.

[21] Tong X, Cui L, Zhao Y (2004). Macromolecules.

[22] Ma Y, Cheng X, Ma H, He Z, Zhang Z, Zhang W (2022). Chem Sci.

[23] Min P, Li Y, Wang L, Song W, Ding L (2022). Polymer.

[24] Ho M S, Natansohn A, Rochon P (1995). Macromolecules.

[25] Hestand N J, Spano F C (2018). Chem Rev.

[26] Ma S, Du S, Pan G, Dai S, Xu B, Tian W (2021). Aggregate.

[27] Kohn W, Becke A D, Parr R G (1996). J Phys Chem.

[28] Tsuneda T, Hirao K (2014). Wiley Interdiscip Rev: Comput Mol Sci.

[29] Ditchfield R, Hehre W J, Pople J A (1971). J Chem Phys.

[30] Hehre W J, Ditchfield R, Pople J A (1972). J Chem Phys.

[31] Marenich A V, Cramer C J, Truhlar D G (2009). J Phys Chem B.

[32] (2016). Gaussian 09.

[33] (2018). The PyMol Graphics System.

