# Supplementary material for: Structure-dependent thermochromism of PAZO thin films: theory and experiment
Source: Beilstein J Nanotechnol. 2026 Jan 20;17:186–99. doi: 10.3762/bjnano.17.12 (PMC12834053; doi:10.3762/bjnano.17.12)
Supplement: File 1 — Additional experimental data. [file Beilstein_J_Nanotechnol-17-186-s001.pdf]

BEILSTEIN JOURNAL OF NANOTECHNOLOGY

Supporting Information

for

Structure-dependent thermochromism of PAZO thin films: theory and experiment

Georgi Mateev, Dean Dimov, Nataliya Berberova-Buhova, Nikoleta Kircheva, Todor Dudev, Ludmila Nikolova, Elena Stoykova, Keehoon Hong, Dimana Nazarova, Silvia Angelova and Lian Nedelchev

Beilstein J. Nanotechnol. **2026,** *17,* 186–199. doi:10.3762/bjnano.17.12

Additional experimental data

License and Terms: This is a supporting information file under the terms of the Creative Commons Attribution License (https://creativecommons.org/licenses/by/4.0). Please note that the reuse, redistribution and reproduction in particular requires that the author(s) and source are credited and that individual graphics may be subject to special legal provisions.
The license is subject to the *Beilstein Journal of Nanotechnology* terms and conditions: (https://www.beilstein-journals.org/bjnano/terms)

Differential thermal analysis (DTA) and thermogravimetric analysis (TGA) of PAZO

The study of PAZO was conducted up to a temperature of 300 °C in order to establish the boundary conditions and determine whether heating would induce changes in the optical spectrum. The spectra were recorded in real time at each temperature reached. To achieve this, experiments were performed at different heating rates of 5 °C/min and 1 °C/min, allowing the sample to equilibrate at the slower rate.

From the DTA and TGA analyses (Figure S1), a noticeable change in weight and in the rate of weight loss is observed at around 281 °C, where the steepest slope of the thermogravimetric curve occurs. These changes also produced a distinct optical response, manifested as a blue shift of the absorption peaks.

Based on these observations, 230 °C was selected as the operating temperature corresponding to the maximum optical response, which coincides with a 6% weight loss and lies within the linear region of the thermogravimetric curve. No chemical changes are expected to occur in this temperature range.

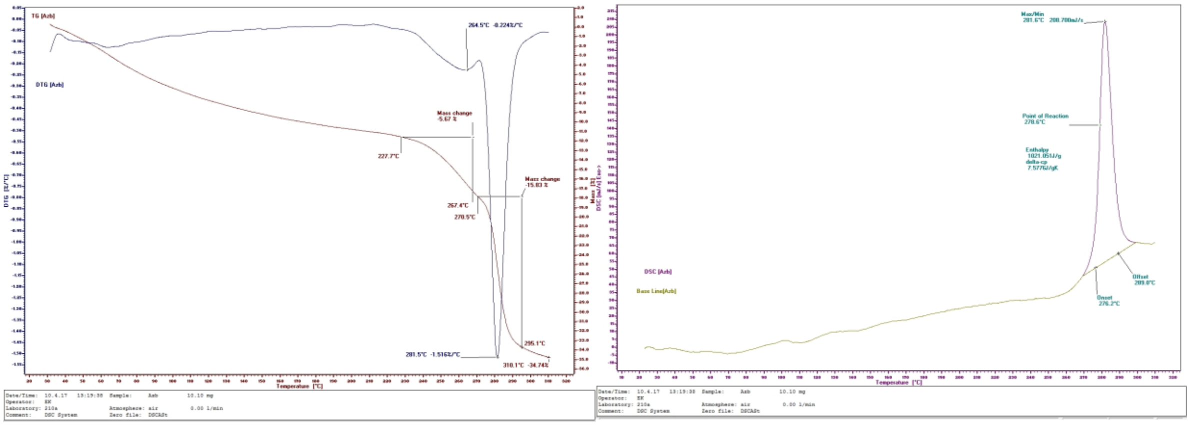

Figure S1: Differential thermal analysis (DTA) and thermogravimetric analysis (TGA) of PAZO.
